# Vitamin D-Binding protein Gene Polymorphism Predicts Pegylated Interferon-Related HBsAg Seroclearance in *HBeAg*-Negative Thai Chronic Hepatitis B Patients: A Multicentre Study

**DOI:** 10.31557/APJCP.2019.20.4.1257

**Published:** 2019

**Authors:** Kessarin Thanapirom, Sirinporn Suksawatamnuay, Wattana Sukeepaisarnjaroen, Sombat Treeprasertsuk, Tawesak Tanwandee, Phunchai Charatcharoenwitthaya, Satawat Thongsawat, Apinya Leerapun, Teerha Piratvisuth, Rattana Boonsirichan, Chalermrat Bunchorntavakul, Chaowalit Pattanasirigool, Bubpha Pornthisarn, Supot Tuntipanichteerakul, Ekawee Sripariwuth, Woramon Jeamsripong, Theeranan Sanpajit, Yong Poovorawan, Piyawat Komolmit

**Affiliations:** 1 *Division of Gastroenterology, Department of Medicine, Faculty of Medicine, *; 2 *Center of Excellence in Liver Diseases, King Chulalongkorn Memorial Hospital, *; 3 *Research unit of hepatic fibrosis and cirrhosis, Department of Medicine,*; 16 *Center of Excellence in Clinical Virology, Department of Pediatrics, Faculty of Medicine, Chulalongkorn University, Thai Red Cross Society,*; 5 *Division of Gastroenterology, Department of Medicine, Siriraj Hospital*; 8 *Faculty of Medicine, Vajira Hospital,*; 9 *Faculty of Medicine, Rajavithi Hospital, *; 10 *Faculty of Medicine, Police General Hospital,*; 11 *Faculty of Medicine, Thammasat University Hospital,*; 12 *Faculty of Medicine, Bhumibol Adulyadej Hospital, *; 15 *Phramongkutklao Hospital, Bangkok,*; 4 *Department of Medicine, Faculty of Medicine, Khon Kaen University, Khon Kaen, *; 6 *Department of Internal Medicine, Chiang Mai University, Chiang Mai,*; 7 *Faculty of Medicine, Prince of Songkla University, Songkhla, *; 13 *Faculty of Medicine, Naresuan University,*; 14 *Faculty of Medicine, Buddhachinaraj Hospital, Phitsanulok, Thailand. *

**Keywords:** Vitamin D, polymorphisms, response, pegylated-interferon, HBeAg-negative, Hepatitis B virus infection

## Abstract

**Background:**

Vitamin D deficiency is related to poor clinical outcomes in patients with chronic hepatitis B virus (HBV) infection.

**Methods:**

We aimed to investigate the association between the genetic variants in the vitamin D metabolic pathway and the response to pegylated interferon (Peg-IFN) therapy in patients with HBeAg-negative chronic HBV infection. One hundred seven patients treated with Peg-IFN for 48 weeks were selected from 13 specialty hospitals. Eight genotypes of vitamin D cascade genes, including *CYP27B1* (rs10877012), *DHCR7* (rs12785878), *CYP2R1* (rs2060793, rs12794714) and *GC* (rs4588, rs7041, rs222020, rs2282679), were found.

**Results:**

Eighty-two patients (83.7%) were infected with HBV genotype C. Eight patients had compensated liver cirrhosis (8.7%). At 24 weeks after treatment discontinuation, 41 patients (42.3%) achieved sustained treatment response, 53 (55.2%) obtained HBV DNA<2,000 IU/ml, 6 (5.6%) gained HBsAg seroclearance, 2 (1.9%) had HBsAg seroconversion and 69 (64.5%) exhibited alanine aminotransferase (ALT) normalization. Multivariate analysis revealed that baseline HBsAg level (OR =0.06, 95% CI: 0.08-0.49, p=0.008) and the *GC rs222020 TT* genotype (OR=17.72, 95% CI: 1.07-294.38, p=0.04) independently predicted sustained HBsAg seroclearance. In addition, this genotype was a predictor for normalization of ALT (OR=4.61, 95%CI: 1.59-13.40, p=0.005) after therapy. The HBsAg levels at baseline and during and post-treatment tended to be reduced with the *GC rs222020 TT* compared with the non-TT genotypes. The other studied polymorphisms were not associated with treatment response.

**Conclusions:**

The *GC rs222020 TT* genotype, which is a variant in the vitamin D-binding protein gene, could identify HBeAg-negative patients who have a high probability to achieve HBsAg clearance and ALT normalization after treatment with Peg-IFN.

## Introduction

Hepatitis B virus (HBV) infection represents a public global health problem with approximately 250 million people were chronically infected (Schweitzer et al., 2015). Chronic HBV infection is the major contributor to the development of cirrhosis, liver failure, hepatocellular carcinoma and liver-related death worldwide (Liaw and Chu, 2009). Currently, two approved therapeutic strategies are available, including Pegylated interferon (Peg-IFN) and nucleos(t)ide analogues (NAs), which suppress HBV replication and slow disease progression; however, these treatments do not generally achieve a cure (Lok et al., 2016; Ghany, 2017). The ultimate endpoint of chronic HBV treatment is sustained HBsAg loss with or without seroconversion to hepatitis B surface antibody (anti-HBs). Treatment with Peg-IFN results in increased HBsAg seroclearance compared with NAs, but it is associated with numerous adverse effects. Therefore, selecting patients who have a high chance to achieve a treatment response to Peg-IFN is essential.

Vitamin D is an important emerging immunomodulator for both innate and adaptive immune responses (von Essen et al., 2010; Prietl et al., 2013). Increasing evidence confirms that vitamin D deficiency is highly prevalent among patients with chronic liver disease (64-92%) and inversely associated with severity of disease (Fisher and Fisher, 2007; Arteh et al., 2010; Kim et al., 2017). In patients with chronic hepatitis C infection, adding vitamin D improves sustained virological response to Peg-IFN and ribavirin therapy and reverses serum marker of hepatic fibrogenesis (Nimer and Mouch, 2012; Yokoyama et al., 2014; Komolmit et al., 2017). Vitamin D deficiency is potentially associated with high HBV replication, poor clinical outcomes and poor virological response to telbivudine-based therapy in chronic HBV patients (Farnik et al., 2013; Wong et al., 2015; Hoan et al., 2016; Yu et al., 2017). The association between common single nucleotide polymorphisms (SNPs) in the vitamin D synthetic pathway, including *CYP27B1*, *DHCR7*, *CYP2R1* and *GC*, and serum vitamin D concentration has been reported in previous genome-wide association studies and systematic reviews (Ahn et al., 2010; Bu et al., 2010; McGrath et al., 2010; Wang et al., 2010). We aim to evaluate the association between genetic polymorphisms in the vitamin D synthesis pathway and response to treatment with Peg-IFN in HBeAg-negative chronic HBV patients.

## Material and Methods

*Patient population*

We retrospectively enrolled Thai patients with HBeAg-negative chronic HBV infection who were treated with 180 µg Peg-IFN alfa-2a (Pegasys, Roche Holding AG, Switzerland) once weekly for 48 weeks between 2010 and 2011 from the hepatitis B database of the Thai Association for the Study of the Liver (THASL). The patients were included from 13 tertiary referral centres in Thailand.

Patients were eligible for this study if they had been HBsAg seropositive for at least 6 months, seronegative for HBeAg, exhibited persistent ALT elevation and HBV DNA > 2,000 IU/mL. Exclusion criteria included previous treatment with Peg-IFN or NAs; co-infection with human immunodeficiency virus, hepatitis C virus, or hepatitis D virus; alcohol abuse; decompensated liver cirrhosis; or contraindicated for Peg-IFN injection. 

*Data collection*

Patient baseline and demographic data were collected before treatment. HBV DNA, HBsAg level, anti-HBs and ALT level were assessed before, every 3 months during treatment, and at 24 weeks after the completion of therapy. Liver cirrhosis was diagnosed by liver biopsy, radiological examination or transient elastography (Fibroscan®, Echosens, Paris, France). 

The study protocol was approved by the Institutional Review Boards of the Faculty of Medicine, Chulalongkorn University (536/54), Faculty of Medicine, Khon Kaen University (HE551219), Faculty of Medicine Mahidol University (562/2555), Faculty of Medicine Chiang Mai University (MED-2556-01570), Faculty of Medicine, Prince of Songkla University (56-041-14-1-2), Faculty of Medicine, Vajira Hospital (090/55), Faculty of Medicine, Rajavithi Hospital, Faculty of Medicine, Police General Hospital (64/2555), Faculty of Medicine, Thammasat University (MTU-EC-1M-4-105/55), Faculty of Medicine, Bhumibol Adulyadej Hospital, Faculty of Medicine, Naresuan University (55 02 04 0006), Faculty of Medicine, Buddhachinaraj Hospital (105/55), Faculty of Medicine, Phramongkutklao Hospital (S042h/52) and conducted in compliance with the principles of the Declaration of Helsinki. Informed consent have been obtained from all of the participants.

*Definition of treatment responses*

The definition of treatment response in this study was defined based on the most recent guidelines for management chronic HBV infection (Sarin et al., 2016; Terrault et al., 2016; European Association for the Study of the Liver. Electronic address and European Association for the Study of the, 2017). Sustained response was defined as serum HBV DNA < 2,000 IU/ml combined with ALT normalization at 24 weeks after completion of Peg-IFN treatment. Virological response was defined as serum HBV DNA < 2,000 IU/ml. HBsAg seroclearance was defined as undetectable HBsAg (HBsAg < 0.05 IU/ml). ALT normalization was defined as a reduction of serum ALT to the normal range (< 30 U/L).

*Laboratory assays*

Qualitative HBsAg, anti-HBs, HBeAg and anti-HBe were assessed using electrochemiluminescence immunoassays (Roche Diagnostics, Indianapolis, IN, USA or Architect, Abbott Diagnostics, Abbot Park, IL, USA). HBsAg levels were assessed using Elecsys HBsAg II Quant reagent kits (Roche Diagnostics, Indianapolis, IN, USA). HBV DNA was quantified using the real time PCR COBAS AmpliPrep-COBAS TaqMan HBV test (Roche Molecular Systems, NJ, Branchburg, USA). HBV genotype was evaluated using INNO-LiPA line probe assay (Innogenetics, Ghent, Belgium).

*Genotyping of the studied polymorphisms*

Genomic DNA was extracted from blood samples using phenol-chloroform. The polymerase chain reaction (PCR) was performed, followed by restriction fragment length polymorphism assays, to detect eight studied polymorphisms in the vitamin D pathway, including *CYP27B1* (rs10877012 C > A), *DHCR7* (rs12785878 G > T), *CYP2R1* (rs2060793 T > C, rs12794714 C > T) and *GC* (rs4588 C > A, rs7041 G >T, rs222020 C > T, rs2282679 A > C). The specific primer sequences and PCR conditions are presented in supplement Table 1. The resulting DNA fragments were separated by electrophoresis on 2% agarose gel after staining with ethidium bromide.

*Statistical analysis*

Statistical analysis was conducted using SPSS software, version 22.0 (IBM Corp, Armonk, NY, USA). Categorical variables are presented as frequency (%) and compared using the chi-square (χ^2^) or Fisher’s exact test. Continuous variables are summarized as the median (range) and compared using the Mann–Whitney U-test or Student’s t-test. Multiple logistic regression analysis was used to identify predictors for treatment response. Statistical significance was considered as a two-sided P-value < 0.05. Bonferroni correction was used to adjust P-value when several comparisons were performed on a single factor. The chi-square (χ^2^) test was employed to evaluate whether the studied genetic polymorphisms were distributed in Hardy-Weinberg equilibrium (HWE).

## Results

*Patient characteristics*

A total of 107 patients with HBeAg-negative chronic HBV infection were included. The mean age of study participants was 47.0 ± 9.5 years, and most were male (76.6%) and had body mass index < 25 kg/m^2^ (57%). The HBV genotype distribution was A 1% (n = 1), B 15.3% (n = 15), and C 83.7% (n = 82). Eight patients (8.7%) exhibited compensated liver cirrhosis. The median baseline HBV DNA, HBsAg level and ALT were 5.8 log10IU/ml, 3.3 log10IU/ml and 74 U/L, respectively. Baseline characteristics of patients based on HBsAg seroclearance at 24 weeks after treatment completion is summarized in Table 1. HBeAg-negative patients who achieved sustained HBsAg seroclearance after Peg-IFN therapy exhibited reduced pretreatment HBsAg levels compared with those who achieved sustained HBsAg persistence.

Prevalence of *CYP27B1*, *DHCR7*, *CYP2R1* and *GC* polymorphisms and their association with response to Peg-IFN treatment

At the end-of-treatment with Peg-IFN therapy, 81.8% (n = 72) achieved HBV DNA < 2,000 IU/ml, 4.7% (n = 5) exhibited HBsAg loss and 64.5% (n = 69) exhibited ALT normalization. In addition, 42.3% (n = 41) achieved sustained treatment response, 55.2% (n = 53) obtained HBV DNA < 2,000 IU/ml, 5.6% (n = 6) had HBsAg seroclearance, 1.9% (n = 2) achieved HBsAg seroconversion and 64.5% (n = 69) exhibited ALT normalization at 24 weeks following treatment discontinuation. 

The genotypic frequencies of *CYP27B1* (rs10877012), *DHCR7* (rs12785878), *CYP2R1* (rs2060793, rs12794714) and *GC* (rs4588, rs7041, rs222020, rs2282679) variants in the vitamin D pathway in patients with HBeAg-negative chronic HBV infection and their association with HBsAg seroclearance at 24 weeks after Peg-IFN treatment is presented in Table 2. All genotypes were in HWE (p>0.05). Sustained HBsAg loss was significantly increased in patients with the TT genotype of *GC* rs222020 (15.2%) compared with patients with non-TT genotypes (1.4%) (p = 0.004). The T-allele of *GC* rs222020 was more frequently noted in patients who gained HBsAg loss compared with those who did not (84% vs. 50%, p<0.05). Additionally, this polymorphism was related with sustained ALT normalization after therapy. Patients with the *GC* rs222020 TT genotype were more likely to achieve ALT normalization (84.8%) at 24 weeks after completion of therapy compared with those who harbour non-TT genotypes (55.4%) (p = 0.003). Table 3 presents baseline characteristics of patients with HBeAg-negative chronic HBV infection with the TT and non-TT genotypes of *GC* rs222020. Patients with the *GC* rs222020 TT genotype (60.6%) included fewer males compared with patients with non-TT genotypes (83.8%) (p = 0.009). No association was noted between the remaining studied SNPs and HBsAg seroclearance, sustained treatment response, virological response or ALT normalization after 24 weeks of follow-up (Supplement Table 2). 

*Baseline predictors for response of treatment at 24 weeks after treatment completion*

To identify baseline predictors associated with treatment response, potentially confounding factors obtained from univariate analysis with p ≤ 0.1 were evaluated by multivariate logistic regression analysis. The results of univariate and multivariate analysis of baseline predictors and sustained HBsAg seronegative were shown in Table 4. In the univariate analysis, low pretreatment HBsAg level, *GC* rs4588 non-CC genotype and *GC* rs222020 TT genotype were associated with sustained HBsAg loss. However, following multivariate analysis, pretreatment HBsAg level (OR = 0.06, 95% CI: 0.08-0.49, p = 0.008) and the *GC* rs222020 TT genotype (OR = 17.72, 95% CI: 1.07-294.38, p = 0.04) were independent predictors of HBsAg seroclearance in response to Peg-IFN treatment. In addition, pretreatment HBsAg level was identified as a predictor of virological response (OR = 0.40, 95% CI: 0.17-0.95, p = 0.037). The *GC* rs222020 TT genotype was a predictor of sustained ALT normalization (OR = 4.51, 95% CI: 1.57-12.96, p = 0.003). No factor was associated with sustained treatment response at 24 weeks post-therapy.

*HBV DNA and HBsAg level kinetics during treatment according to GC rs222020*

**Figure 1:**
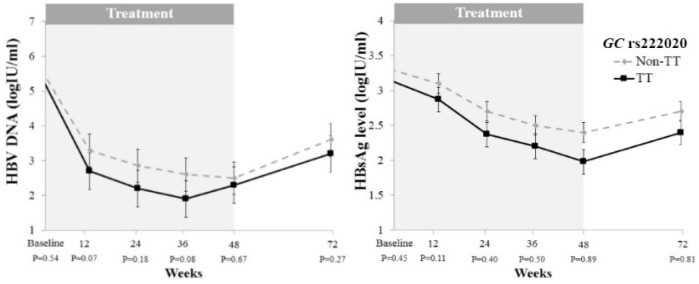
Serum HBV DNA (A) and Serum HBsAg Levels (B) at Baseline, During Treatment and Post-Treatment in Patients with HBeAg-Negative Chronic HBV Infection

The kinetics of HBV DNA and HBsAg levels at baseline, during and after Peg-IFN therapy based on the *GC* rs222020 genotype are presented in Figure 1. A p-value < 0.008 based on Bonferroni adjustment was determined to be statistically significant. In HBeAg-negative chronic HBV patients, the *GC* rs222020 TT genotype tended to be associated with reduced HBV DNA and HBsAg levels at baseline, during and after treatment compared with non-TT genotypes. However, no statistically significant differences were noted.

In terms of early HBsAg decline, patients with the *GC* rs222020 TT genotype were more likely to exhibit a serum HBsAg reduction of ≥ 0.5 log10IU/ml (40%, n = 12) compared with non-TT genotypes (20.3%, n = 12) (p = 0.04) at week 12 of Peg-IFN therapy. Mean levels of serum HBsAg decreased from baseline to end of treatment did not differ between the *GC* rs222020 TT and non-TT genotypes (1.18 ± 1.26 vs. 0.87 ± 1.03 log10IU/ml, p = 0.38).

## Discussion

The major finding of the present study was that the *GC* rs222020 TT genotype and baseline HBsAg level predicted sustained HBsAg seroclearance in HBeAg-negative patients chronic HBV infection treated with Peg-IFN. Furthermore, the *GC* rs222020 TT genotype was independently associated with ALT normalization after completing Peg-IFN therapy. To our knowledge, this is the first study to demonstrate the association between the *GC* polymorphism in vitamin D synthetic pathway and the outcome at 24 weeks after Peg-IFN treatment in patients with HBeAg-negative chronic hepatitis B infection.

Currently, HBsAg seroclearance is considered as the optimal therapeutic endpoint, which represents the closest point to a functional cure. HBsAg seroclearance is associated with very low viremic phase and suppression of HBV replication, resulting in a reduction of long-term complications (Yuen et al., 2008). Treatment with Peg-IFN has a finite duration with an absence of HBV resistance and achieves greater HBsAg loss (3-7%) compared with treatment with NAs (0-3%) (Marcellin et al., 2004; Lau et al., 2005). Two mechanisms of action of Peg-IFN for controlling HBV infection are directly inhibiting HBV DNA synthesis and increasing the cellular immune response against infected hepatocytes (Micco et al., 2013). However, Peg-IFN therapy is affected by limited tolerability according to its adverse effects and contraindications, such as decompensated cirrhosis and co-morbidities (Terrault et al., 2016; European Association for the Study of the Liver. Electronic address and European Association for the Study of the, 2017). Patients should be carefully selected for this treatment based on their probability of response (Konerman and Lok, 2016). Monitoring of serum HBsAg and HBV DNA during therapy have been widely used for Peg-IFN discontinuation. In Thai patients with HBeAg-negative treated with Peg-IFN, the performance of early on treatment stopping rules for sustained response, including no decline in HBsAg level with a <2log_10_ decline in HBV DNA, and <10% decline in HBsAg level at 12-week during therapy showed negative predictive values of 80% and 66%, respectively. Several favourable pre-treatment predictors of response to Peg-IFN have been reported, including low HBV DNA, low HBsAg level, high ALT levels and HBV genotype (Buster et al., 2009; Wang et al., 2016). Previous studies have revealed the association between host genetic variants and Peg-IFN-related HBsAg seroclearance in HBeAg-negative chronic HBV patients, including *IL28B* and *CYP27B1* (de Niet et al., 2012; Lampertico et al., 2013; Boglione et al., 2014; Boglione et al., 2015). Nevertheless, the results remain debated and are inconclusive. Further studies are needed to prove this relation.

**Table 1:** Baseline Characteristics of Patients with HBeAg-Negative Chronic Hepatitis B Infection Based on HBsAg Response at 24-Week Follow-up

	HBsAg persistence (n=101)	HBsAg loss (n=6)	p value
Male, n (%)	78 (77.2%)	4 (66.7%)	0.55
Age (years), mean ± SD	47.0 ± 9.7	47.3 ± 6.3	0.94
Body mass index (kg/m^2^), mean ± SD	24.4 ± 3.6	25.2 ± 1.8	0.59
HBV genotype, n (%)			
A	0 (0%)	1 (16.7%)	
B	15 (16.3%)	0	
C	77 (83.7%)	5 (83.3%)	0.98
Cirrhosis, n (%)	7 (8%)	1 (20%)	0.36
Pre-treatment HBV DNA (log_10_IU/ml), mean ± SD	5.61 ± 1.53	4.78 ± 1.58	0.26
Pre-treatment HBsAg level (log_10_IU/ml), mean ± SD	3.28 ± 0.56	2.46 ± 0.49	0.001
Pre-treatment ALT (U/L), mean ± SD	92.8 ± 84.5	60.6 ± 23.0	0.47

**Table 2:** Genotypic Distributions of *CYP27B1, DHCR7, CYP2R1* and *GC* Polymorphisms and Relation with HBsAg Seroclearance after Peg-IFN Therapy in Patients with HBsAg-Negative Chronic HBV Infection

	All patients	HBsAg persistence (n=101)	HBsAg loss (n=6)	Odd ratio (95%CI)	p-value
*CYP27B1* rs10877012 C>A					
CC	28 (26.2%)	25 (24.8%)	3 (50%)	3.04 (0.58-16.03)	0.17
CA	50 (46.7%)	48 (47.5%)	2 (33.3%)		
AA	29 (27.1%)	28 (27.7%)	1 (16.7%)		
*DHCR7* rs12785878 G>T					
GG	56 (52.3%)	52 (51.5%)	4 (66.7%)	1.89 (0.33-10.75)	0.47
GT	40 (37.4%)	38 (37.6%)	2 (33.3%)		
TT	11 (10.3%)	11 (10.9%)	0		
*CYP2R1* rs2060793 T>C					
TT	8 (7.5%)	8 (7.9%)	0	0.94 (0.89-0.99)	0.47
TC	45 (42.1%)	42 (41.6%)	3 (50.0%)		
CC	54 (50.5%)	51 (50.5%)	3 (50.0%)		
*CYP2R*1 rs12794714 C>T					
CC	41 (38.3%)	38 (37.6%)	3 (50%)	1.66 (0.32-8.63)	0.55
CT	53 (49.5%)	51 (50.5%)	2 (33.3%)		
TT	13 (12.1%)	12 (11.9%)	1 (16.7%)		
*GC* rs4588 C>A					
CC	71 (66.4%)	69 (68.3%)	2 (33.3%)	0.23 (0.04-1.33)	0.08
CA	31 (29.0%)	28 (27.7%)	3 (50.0%)		
AA	5 (4.7%)	4 (4.0%)	1 (16.7%)		
*GC* rs7041 G>T					
GG	12 (11.2%)	11 (10.9%)	1 (16.7%)	1.64 (0.17-15.3)	0.66
GT	50 (46.7%)	46 (45.5%)	4 (66.7%)		
TT	45 (42.1%)	44 (43.6%)	1 (16.7%)		
*GC* rs222020 C>T					
CC	28 (26.2%)	28 (27.7%)	0		
CT	46 (43.0%)	45 (44.6%)	1 (16.7%)		
TT	33 (30.8%)	28 (27.7%)	5 (83.3%)	13.04 (1.46-116.57)	0.004
*GC* rs2282679 A>C					
AA	68 (63.6%)	66 (65.3%)	2 (33.3%)	0.26 (0.05-1.52)	0.11
AC	33 (30.8%)	30 (29.7%)	3 (50.0%)		
CC	6 (5.6%)	5 (5.0%)	1 (16.7%)		

**Table 3:** Baseline Characteristics of Patients with HBeAg-Negative Chronic HBV Infection Based on the *GC* rs222020 Variant

	*GC* rs222020 C>T		p value
	CC/CT (n=74)	TT (n=33)	
Male, n (%)	62 (83.8%)	20 (60.6%)	0.009
Age (years), mean ± SD	47.4 ± 10.1	46.2 ± 8.2	0.54
Body mass index (kg/m^2^), mean ± SD	24.6 ± 3.6	24.3 ± 3.2	0.71
HBV genotype C, n (%)	60 (87.0%)	22 (75.9%)	0.17
Cirrhosis, n (%)	4 (6.3%)	4 (14.3%)	0.21
Pre-treatment HBV DNA (log_10_IU/ml), mean ± SD	5.6 ± 1.6	5.5 ± 1.4	0.54
Pre-treatment HBsAg level (log_10_IU/ml), mean ± SD	3.3 ± 0.6	3.2 ± 0.6	0.45
Pre-treatment ALT (U/L), mean ± SD	93.2 ± 89.2	86.7 ± 66.8	0.88

**Table 4:** Univariate and Multivariate Analysis between Baseline Factors and Sustained HBsAg Loss at 24 Weeks after Peg-IFN Discontinuation

	Univariate analysis		Multivariate analysis	
	OR (95% CI)	p-value	OR (95%CI)	p-value
male (vs. female)	0.59 (0.10-3.43)	0.55		
Age, years	1.04 (0.92-1.10)	0.94		
Body mass index (kg/m^2^)	1.06 (0.85-1.33)	0.59		
HBV genotype (C vs. non-C)	0.97 (0.11-8.94)	0.98		
Cirrhosis (yes vs. no)	2.86 (0.28-29.18)	0.36		
HBV DNA (log_10_IU/ml)	0.70 (0.39-1.28)	0.25		
HBsAg level (log_10_IU/ml)	0.13 (0.03-0.51)	0.004	0.06 (0.008-0.49)	0.008
ALT (U/L)	0.99 (0.97-1.01)	0.39		
*CYP27B1 *rs10877012 (CC vs. CA/AA)	3.04 (0.58-16.03)	0.17		
*DHCR7* rs12785878 (GG vs. GT/TT)	1.89 (0.33-10.75)	0.47		
*CYP2R1 *rs2060793 (TT vs. TC/CC)	0.94 (0.89-0.99)	0.47		
*CYP2R1* rs12794714 (CC vs. CT/TT)	1.66 (0.32-8.63)	0.55		
*GC* rs4588 (CA/AA vs. CC)	4.31 (0.75-24.78)	0.08	2.16 (0.22-21.27)	0.51
*GC* rs7041 (GG vs. GT/TT)	1.64 (0.17-15.3)	0.66		
*GC* rs222020 (TT vs. CC/CT)	13.04 (1.46-116.57)	0.004	17.72 (1.07-294.38)	0.04
*GC* rs2282679 (AA vs. AC/CC)	0.26 (0.05-1.52)	0.11		

Over the last decades, the effects of vitamin D on immune response have been described, including descriptions of the expression of the vitamin D receptor (VDR) or vitamin D-activating enzymes involved in the activation and differentiation of immune cells (Prietl et al., 2013). In patients with chronic HBV infection, the prevalence of vitamin D deficiency was more common (13.3%) compared with healthy controls (1.6%) (Chen et al., 2015). Vitamin D deficiency is associated with high levels of HBV replication, high Child-Pugh scores, poor adverse clinical outcomes and poor response to Peg-IFN therapy (Farnik et al., 2013; Chen et al., 2015; Zhao et al., 2016; Yu et al., 2017). The vitamin D level and the genetic variation in the vitamin D cascade might be related with treatment outcome. A prior study demonstrated the association between the TT genotype of *CYP2R1* rs12794714 polymorphism and sustained HBeAg seroconversion in HBeAg-positive chronic HBV patients treated with Peg-IFN (Thanapirom et al., 2017). In patients with HBeAg-negative chronic HBV infection, the *CYP27B1* rs4646536 TT genotype and the VDR Apa- AA genotype were associated with sustained virological response after Peg-IFN therapy (Boglione et al., 2015; Cusato et al., 2017). 

The *GC* gene located on the long arm of chromosome 4 and encodes vitamin D-binding protein, which is responsible for vitamin D transportation, storage and metabolism (Cooke and David, 1985). This gene is expressed predominantly in the liver (Speeckaert et al., 2006). Some *GC* polymorphisms affect serum vitamin D levels. The *GC* rs7041 and rs4588 variants are the most commonly reported SNPs for reduced vitamin D levels in serum and target tissues in different populations (McGrath et al., 2010; Powe et al., 2013). The rs222020 variant is located in the intron region of *GC* and affects serum vitamin D levels in healthy Caucasian subjects and northeastern Han Chinese children (Zhang et al., 2012; Zhang et al., 2013b). The *GC* rs222020 variant is related to the risk of rickets (Zhang et al., 2013a). Additionally, the HALT-C study followed 692 chronic HCV patients after Peg-IFN therapy over 4 years, and the *GC* rs222020 variant was associated with worsening liver fibrosis (de Azevedo et al., 2017). However, this study did not investigate the effect of the *GC* variants on the virological response after treatment. In HBeAg-positive chronic HBV patients, the *GC* rs222020 polymorphism was not related with treatment response to Peg-IFN therapy (Thanapirom et al., 2017). No prior studies have assessed the association between *GC* gene polymorphisms and treatment response in HBeAg-negative chronic HBV patients. Our study demonstrated that the *GC* rs222020 TT genotype predicted sustained HBsAg seroclearance and ALT normalization after completion of Peg-IFN therapy. The mechanism was obscure. One possible hypothesis is that HBeAg-negative patients with the *GC* rs222020 TT genotype tended to exhibit reduced HBsAg levels at baseline, during treatment and post-treatment compared with those with non-TT genotypes. In particular, the TT genotype was associated with an early serum HBsAg reduction that is correlated with a favourable treatment outcome after Peg-IFN therapy (Moucari et al., 2009). This genetic factor might be useful for predicting treatment response and selection of suitable patients for Peg-IFN therapy. Information on prevalence of the *GC* rs222020 genotype in the Thai population was not previously available. However, the prevalence of CC, CT and TT genotype was 14-22%, 41-42% and 37-44%, respectively, in a Japanese and Han Chinese population based on the international Haplotype Map (HapMap) project database. The present study demonstrated that the prevalence of the *GC* rs222020 in the cohort of Thai HBeAg-negative chronic HBV patients was 26.2% for the CC genotype, 43% for CT and 30.8% for TT.

Our study has some limitations. Firstly, even though vitamin D levels might give more valuable information, the baseline vitamin D levels were not evaluated in this study because these levels are affected by several confounding factors, including sunlight exposure, season, dietary intake, skin pigmentation, bowel absorption and severity of liver disease (Moucari et al., 2009). In addition, there is a study suggesting that the baseline serum vitamin D level is not associated with HBsAg loss after Peg-IFN treatment in chronic HBV patients (Chan et al., 2015). For these reasons, we aim to investigate the influence of the functional genetic variants that affect the vitamin D level, which might overcome some of these limitations. This might be the interesting novel strategy for selection patients to treat with Peg-IFN. Secondly, this study had a short period of follow up and included a small number of HBeAg-negative patients. Longer follow-up period might have additional patients with HBsAg seroclearance. However, the number is similar to previous cohort studies (Brunetto et al., 2009; Moucari et al., 2009; Peng et al., 2012; Lampertico et al., 2013; Goulis et al., 2015; Wang et al., 2016). It is of interest that these SNPs might influent more on the long-term treatment outcome, in view of HBsAg seroclearance, a further study is required.

In conclusion, among genetic variants in the vitamin D-binding protein, the *GC* rs222020 TT genotype was an independent predictor for sustained HBsAg seroclearance and normalization of ALT following Peg-IFN therapy in patients with HBeAg-negative chronic HBV infection. This genetic variant could represent a useful pretreatment factor for the selection of candidate patients for Peg-IFN treatment. 

## Funding Statement

The research funding was supported from the Thai Association for the Study of the Liver (THASL), the Ratchadaphiseksomphot Endowment Fund of hepatic fibrosis and cirrhosis research unit (GRU 6105530009-1), and the Ratchadaphiseksomphot Endowment Fund (RA59/074, RA60/101), Chulalongkorn University. Part or partial of the laboratory work was supported by the research Chair Grant (P-15-50004) and the Center of Excellence in Clinical Virology at Chulalongkorn University and Chulalongkorn Hospital (GLE 58-014-30-004, RES560530093).
